# Supplementary material for: Determinants of short birth interval among ever married reproductive age women: A community based unmatched case control study at Dessie city administration, Northern Ethiopia
Source: PLoS One. 2020 Dec 4;15(12):e0243046. doi: 10.1371/journal.pone.0243046 (PMC7717527; doi:10.1371/journal.pone.0243046)
Supplement: S1 Questionnaire — (DOCX) [file pone.0243046.s001.docx]

# S1 Questionnaire: English Questionnaire

Part I: Socio-demographic Characteristics of the respondents on determinants of short birth among ever married reproductive age mothers in Dessie city administration, Dessie, Ethiopia 2019.

House code

| S.N | Questions | Response | Coding | Skip  to Q |
| --- | --- | --- | --- | --- |
| 101 | Residence | Urban | 1 |  |
|  |  | Rural | 2 |  |
| 102 | Age at delivery of the last child |  |  |  |
| 103 | Current marital status | Married | 1 |  |
|  |  | Divorced | 2 |  |
|  |  | Widowed | 3 |  |
|  |  | Others | 4 |  |
| 104 | Number of wives in the household? | 1. One | 1 |  |
|  |  | 2. more than one | 2 |  |
| 105 | At what age did you marry? |  |  |  |
| 106 | Religion | Orthodox . | 1 |  |
|  |  | Muslim | 2 |  |
|  |  | Protestant | 3 |  |
|  |  | Others(specify) | 4 |  |
| 107 | Ethnicity | Amhara | 1 |  |
|  |  | Tigrai | 2 |  |
|  |  | Oromo | 3 |  |
|  |  | Others (specify) | 4 |  |
| 108 | Education of the mother at previous to last birth | No formal education | 1 |  |
|  |  | Able to read and write | 2 |  |
|  |  | Elementary (1 - 8) | 3 |  |
|  |  | Secondary (9 - 12) | 4 |  |
|  |  | Collage and above | 5 |  |
| 109 | Education of the husband | No formal education | 1 |  |
|  |  | Able to read and write | 2 |  |
|  |  | Elementary (1 - 8) | 3 |  |
|  |  | Secondary (9 - 12) | 4 |  |
|  |  | Collage and above | 5 |  |
| 110 | Occupation of the mother at previous to last birth | Employee (GO/NGO) | 1 |  |
|  |  | House wife | 2 |  |

|  |  | Merchant | 3 |  |
| --- | --- | --- | --- | --- |
|  |  | Student | 4 |  |
|  |  | Farmer | 5 |  |
|  |  | Daily worker | 6 |  |
|  |  | Others (Specify) | 7 |  |
| 111 | Occupation of the husband | Employee (GO/NGO) | 1 |  |
|  |  | Merchant | 2 |  |
|  |  | Student | 3 |  |
|  |  | Farmer | 4 |  |
|  |  | Daily worker | 5 |  |
|  |  | Others(specify) | 6 |  |

**Part II:** Knowledge and attitude on Birth Interval on determinants of short birth among ever married reproductive age mothers in Dessie city administration, Dessie, Ethiopia 2019.

| **S.N** | **Questions** | **Response** | **coding** | **Skip** |
| --- | --- | --- | --- | --- |
| **201** | Have you heard about optimal birth interval between two consecutive births? | Yes | **1** | If no go to Q**204** |
|  |  | No | **2** |  |
| **202** | If yes for question 201, From where you heard about optimal birth interval? | Media and press | 1 |  |
|  |  | Health workers | 2 |  |
|  |  | Family | 3 |  |
|  |  | Friends | 4 |  |
|  |  | Others/specify | 5 |  |
| **203** | If yes to question no 201, what is the optimum number of months or years between two successive births? | Below three years | 1 |  |
|  |  | Three to five years | 2 |  |
|  |  | Above five years | 3 |  |
|  |  | I don’t know | 4 |  |
| **204** | Do you think that minimum 3 years of birth spacing is essential between two successive births? | Strongly agree | 1 |  |
|  |  | Agree | 2 |  |
|  |  | No idea | 3 |  |
|  |  | Do not agree | 4 |  |
|  |  | Strongly disagree | 5 |  |
| **205** | What was your husband's beliefs regarding birth spacing? | Disagree strongly | 1 |  |
|  |  | Don't mind | 2 |  |
|  |  | Encouraging | 3 |  |
|  |  | Unknown | 4 |  |

| **206** | Is there family and social influences to practice short birth interval | Yes | 1 |  |
| --- | --- | --- | --- | --- |
|  |  | No | 2 |  |
| **207** | Do you think that adequate/optimum birth spacing has a **healt**h advantages on mother and  child? | Yes | 1 |  |
|  |  | No | 2 |  |
| **208** | Does short birth interval have a  health disadvantages on mother and child? | Yes | 1 |  |
|  |  | No | 2 |  |

**Part III:** obstetrics and Birth History of the respondents on determinants of short birth among ever married reproductive age mothers in Dessie city administration, Dessie, Ethiopia 2019.

| **S.N** | **Questions** | **Response** | **Coding** | **Skip** |
| --- | --- | --- | --- | --- |
| **301** | At what age you had first birth | ----------------- |  |  |
| **302** | How many months wait from marriage to first birth? |  |  |  |
| **303** | What was fetal outcome of first delivery | Live birth | 1 |  |
|  |  | Still birth | 2 |  |
|  |  | Abortion | 3 |  |
|  |  | Neonatal mortality | 4 |  |
| **304** | Have you ever prior history of infertility | Yes | **1** |  |
|  |  | No | 2 |  |
| **305** | How many people live with you? |  |  |  |
| **306** | How many children have you ever born alive? | Males |  |  |
|  |  | Females |  |  |
| **307** | Have you ever given birth to any child who died? | Yes . | 1 | If no go to  Q**312** |
|  |  | No | 2 |  |
| **308** | If yes, how many of your children died? | Males |  |  |
|  |  | Females |  |  |
| **309** | Male to female ratio of living children | More than one | 1 |  |
|  |  | One | 2 |  |
|  |  | Less than one | 3 |  |
|  |  | Males only | 4 |  |
|  |  | Females only | 5 |  |
| **310** | Is previous to last pregnancy is planned | Yes | 1 |  |
|  |  | No | 2 |  |
| **311** | Have you practice postpartum abstinence in before the last child? | Yes | 1 |  |
|  |  | No | 2 |  |
| **312** | If yes duration of postpartum resumption of sexual activity before the last child? |  |  |  |
| **313** | What was mode of delivery of previous to | SVD | 1 |  |

|  | last birth | | | | | | C/S | | | | 2 | |  |
| --- | --- | --- | --- | --- | --- | --- | --- | --- | --- | --- | --- | --- | --- |
|  |  |  |  |  |  |  | instrumental  Delivery | | Vaginal | | 3 | |  |
| **314** | Have you had ANC follow up preceding pregnancy | | | | in | | Yes | | | | 1 | |  |
|  |  |  |  |  |  |  | No | | | | 2 | |  |
| **315** | Where was the place of delivery previous to last birth | | | | of | | Home | | | | 1 | |  |
|  |  |  |  |  |  |  | Health institution | | | | 2 | |  |
| **316** | How was the pattern of menstruation in previous to last deliveries | | | | | | Regular | | | | 1 | |  |
|  |  |  |  |  |  |  | Irregular | | | | 2 | |  |
| **317** | Have you had chronic diseases (HTN,DM  ,others) before the last child?? | | | | | | yes /specify | | | | 1 | |  |
|  |  |  |  |  |  |  | No | | | | 2 | |  |
| **318** | Have you ever history of Postpartum complications in previous to last deliveries | | | | | | Yes | | | | 1 | |  |
|  |  |  |  |  |  |  | No | | | | 2 | |  |
| **319** | If yes What type of complication? (check all that apply) | | | | | | Fever | | | | 1 | |  |
|  |  |  |  |  |  |  | Hemorrhage | | | | 2 | |  |
|  |  |  |  |  |  |  | Seizure or  Preeclampsia | | Headache or | | 3 | |  |
|  |  |  |  |  |  |  | Others | | | | 4 | |  |
| **320**. Birth Order | | **1.** Sex  1. Male 2.  Fema le | **2**. In  what mont h and year did (nam e) born  ? | Birth  orde r | | **3**. Is he  /she aliv e?   1. Yes 2. No | | **4**. If died, how old was (name) he/she died   1. year 2. Month | | **5**.Current age | | **321**. Birth  interval in months | |
| **320.1**.Last  child | |  |  |  | |  | |  | |  | |  | |
| **320.2**.  previous to last  child | |  |  |  | |  | |  | |  | |  | |

**Part IV :**Breast feeding duration on determinants of short birth among ever married reproductive age mothers in Dessie city administration, Dessie, Ethiopia 2019.

| **S.N** | **Questions** | **Response** | **coding** | **Skip** |
| --- | --- | --- | --- | --- |
| **401.** | Did you breast fed previous to last child (name)? | Yes | 1 |  |
|  |  | Never breast fed | 2 |  |

| **402.** | If yes to question 401, for how long was (name) breastfed? | Months |  |  |
| --- | --- | --- | --- | --- |
| **403** | Did you exclusive breastfeeding previous to last child (name) ?, | Yes | 1 |  |
|  |  | No | 2 |  |

**Part V:** Knowledge and practice of modern contraceptive use on determinants of short birth among ever married reproductive age mothers in Dessie city administration, Dessie, Ethiopia 2019.

| **S.N** | **Questions** | **Response** | **Code** | **Skip** |
| --- | --- | --- | --- | --- |
| **501** | Do you know any modern method that women and men can use to delay or avoid pregnancy? | Yes | 1 | If no go to  Q50 4 |
|  |  | No | 2 |  |
| **502** | If yes for q501, which of the following  methods do you know about? |  |  |  |
|  | **502.1**. Pills | 1. Yes 2. No |  |  |
|  | **502. 2**.Injectable | 1. Yes 2. No |  |  |
|  | **502. 3**. Condom | 1. Yes 2. No |  |  |
|  | **502. 4**. Implants | 1. Yes 2. No |  |  |
|  | **502. 5**. IUD | 1. Yes 2. No |  |  |
|  | 502.6 Emergency contraceptive | 1. yes 2. No |  |  |
| **503** | Have you been using any of the modern methods before the conception of your last child? | 1. Yes 2. No |  | If no go to Q**50**  **7** |
|  |  |  |  |  |
| **504** | If yes to question no **504,** which of the following modern methods did you use? | Pills | 1 |  |
|  |  | Injectable | 2 |  |
|  |  | Condom | 3 |  |
|  |  | Implanon | 4 |  |
|  |  | IUCD | 5 |  |
| **505** | Decision maker about Family planning | Self (mother) | 1 |  |
|  |  | Both husband and wife | 2 |  |
|  |  | Husband only | 3 |  |
| **506** | How far do you travel from primary health  center |  |  |  |
| **507** | What is your believes about family planning  method is necessarily to birth spacing | Agree | 1 |  |
|  |  | Disagree | 2 |  |

|  |  | Neutral | 3 |  |
| --- | --- | --- | --- | --- |

**Part VI:** Socio-economic characteristics of respondents on determinants of short birth among ever married reproductive age mothers in Dessie city administration, Dessie, Ethiopia 2019

| **S.N** | **Questions** | **Response** | | | **Coding** | **Skip** |
| --- | --- | --- | --- | --- | --- | --- |
| 601 | Does any member of this household has own any  agricultural land? | Yes | | | 1 |  |
|  |  | No | | | 2 |  |
| 602 | How much (local units) of agricultural land does  members of this household own? | ( in Hectare) | | |  |  |
| 603 | Does this household own any livestock, herds, other  farm animals, or poultry? | Yes | | | 1 | 605 |
|  |  | No | | | 2 |  |
| 604 | How many of the following animals, does this  household own?  **[**PROBE AND MARK THAT ALL APPLY,  MULTIPLE ANSWER IS POSSIBLE] |  | Animals | No |  |  |
|  |  |  | Milk cows, oxen  or bulls |  |  |  |
|  |  |  | Horses, donkey or mules |  |  |  |
|  |  |  | Goet |  |  |  |
|  |  |  | Sheep |  |  |  |
|  |  |  | Chicken |  |  |  |
|  |  |  | Beehives |  |  |  |
| 605 | Which of the following does your household | 1. Electricity | | |  |  |
|  | have? | 2. Watch | | |  |  |
|  | *(Record observation, Multiple response is possible)* | 3. Radio | | |  |  |
|  |  | 4. Television | | |  |  |
|  |  | 5. Mobile Telephone | | |  |  |
|  |  | 6. Non Mobile Telephone | | |  |  |
|  |  | 7. Chair | | |  |  |
|  |  | 8. Table | | |  |  |
|  |  | 9. Bed | | |  |  |
|  |  | 10. Electric Mitad | | |  |  |
|  |  | 11. Other (specify) | | |  |  |
| 606 | What is main current source of drinking water | 1. Piped water | | |  |  |
|  | for | 2. Public Tap/Stand Pipe | | |  |  |
|  | members of your house hold? | 3. Borehole | | |  |  |
|  |  | 4. Protected well | | |  |  |
|  |  | 5. Unprotected well | | |  |  |
|  |  | 6. Protected Spring | | |  |  |
|  |  | 7. Unprotected Spring | | |  |  |
|  |  | 8.River/Ponds/Stream/Dam | | |  |  |
|  |  | 9. Other (specify) _ | | |  |  |

| 607 | What kind of toilet facility do members of your  household usually use?  *(Record Observation)* | 1. Flush to piped sewer system 2. Flush to septic tank 3. Pit latrine with slab 4. Pit latrine without slab 5.Ventilated improved pit latrine 5. No facility/bush/field 6. Other (specify) |  |  |
| --- | --- | --- | --- | --- |
| 608 | What are the main materials of the floor of house?  *(Record observation)* | 1. Earth/Sand 2. Wood planks 3. Palm/Bamboo 4. Ceramic Tiles 5. Cement 6. Other (specify) |  |  |
| 609 | What are the main materials of the roof of house?  *(Record observation)* | 1. Thatch/straw 2. Leaf/Earth/ Mud/Cow dung 3. Wood planks, cardboard 4. Finished roof (iron, tin, finished wood, cement, ceramic) 5. Other(specify) |  |  |
| 610 | What is main material of the exterior walls of house?  *(Record observation)* | 1. Simple wall with mud or local materials 2. Bamboo or stone with mud, plywood, cardboard 3.Finished walls; cement, brick, stone with cement, wood planks 3. No outside walls 4. Others (specify) |  |  |
| 611 | Which means of transport (vehicles) does any member  of your household own? | 1. Bicycle 2. Motor cycle 3. Animal-drawn cart 4. Car/truck 5.Bajag   6. None |  |  |

# Thank you for the interview!!
