# Supplementary material for: Determinants of short birth interval among ever married reproductive age women: A community based unmatched case control study at Dessie city administration, Northern Ethiopia
Source: PLoS One. 2020 Dec 4;15(12):e0243046. doi: 10.1371/journal.pone.0243046 (PMC7717527; doi:10.1371/journal.pone.0243046)
Supplement: Attachment — Submitted filename: wubet 1Response letter for plos (2).docx [file pone.0243046.s002.docx]

**Response letter**

**Dear editor**

*After going through the entire manuscript, you forwarded your constructive editorial comments which we missed to touch. Therefore, we are glad enough to express our sincerest thanks for your in-depth review and comments that could help improve the tone and readability of our paper.*

**Editor comment 1:** Please ensure that your manuscript meets PLOS ONE's style requirements, including those for file naming. The PLOS ONE style templates can be found at https://journals.plos.org/plosone/s/file?id=wjVg/PLOSOne_formatting_sample_main_body.pdf andhttps://journals.plos.org/plosone/s/file?id=ba62/PLOSOne_formatting_sample_title_authors_affiliations.pdf.

**Authors’ response :** Very important comment it is! Thus, we have ensured that our manuscript meets PLOS ONE's style requirements, including those for file naming by finding the aforementioned link for PLOS ONE style templates.

**Editor comment 2:** Please include additional information regarding the survey or questionnaire used in the study and ensure that you have provided sufficient details that others could replicate the analyses. For instance, if you developed a questionnaire as part of this study and it is not under a copyright more restrictive than CC-BY, please include a copy, in both the original language and English, as Supporting Information. In addition, please refrain from stating p values as .000, either state the exact value or use the format p<0.001.

**Authors’ response:** Undoubtedly! There is a need for including the survey or questionnaire in the study. Besides, we have ensured that we have provided sufficient details that others could replicate the analyses as provided in the additional information file. We didn’t develop questionnaire as part of this study and hence no worry about copyright. In addition, we have refrained from stating p values as .000, rather we stated the exact value as it can be noticed from the yellow highlight in the revised version manuscript.

The questionnaire is newly added as additional information as it can be seen from the yellow highlight on pages 7 and 24 of the revised version manuscript.

**Editor comment** 3. PLOS requires an ORCID iD for the corresponding author in Editorial Manager on papers submitted after December 6th, 2016. Please ensure that you have an ORCID iD and that it is validated in Editorial Manager. To do this, go to ‘Update my Information’ (in the upper left-hand corner of the main menu), and click on the Fetch/Validate link next to the ORCID field. This will take you to the ORCID site and allow you to create a new iD or authenticate a pre-existing iD in Editorial Manager. Please see the following video for instructions on linking an ORCID iD to your Editorial Manager account: https://www.youtube.com/watch?v=_xcclfuvtxQ

**Authors’ response:** the corresponding author didn’t have an ORCID iD before. Thus, based on your recommendation, the corresponding author created a new ORCID iD ([0000-0002-5972-4818](javascript:popup_orcidDetail(%22https://orcid.org%22,%20%220%22);)) in the PLOS Editorial Manager.

**Editor comment** 4. Your ethics statement must appear in the Methods section of your manuscript. If your ethics statement is written in any section besides the Methods, please move it to the Methods section and delete it from any other section. Please also ensure that your ethics statement is included in your manuscript, as the ethics section of your online submission will not be published alongside your manuscript.

**Authors’ response:** Absolutely! It is a comment of technical relevance as per the journal requirement. Thus, our ethics statement appears in the methods section of our manuscript. Besides, our ethics statement is deleted from the declaration section and moved to the methods section as shown by the yellow highlighted text on page 8 in the ethical approval and consent to participate subsection, methods section of the revised version manuscript.

Additional Editor Comments (if provided):

Reviewers have returned some substantive comments to improve your manuscript. Please carefully consider each comment and respond appropriately. Please prepare a table indicating how you have responded to each comment and please follow the advice to secure expertise in the correction of English grammar within the script.

**Authors’ response: No doubt! We have tried our best to** carefully consider each reviewer’s comment and respond appropriately. We have also followed the advice to secure expertise in the correction of English grammar within the script. Besides, we have included a point by point response letter as detailed below.

**Dear reviewer 1**

*After going through the entire manuscript, you forwarded your constructive comments which we missed to touch. Therefore, we are glad enough to express our sincerest thanks for your in-depth review and comments that could help improve the tone of ourpaper.*

Reviewer suggestion: The authors have conducted an interesting descriptive analysis of factors that predict birth spacing of < 3 years as opposed to between 3 and 5 years in Ethiopia. They find that contraceptive use, breastfeeding, age at first birth, preceding child sex and underlying knowledge of existing advice regarding birth spacing all influenced the likelihood of a short birth interval. I have several questions for clarification.

**Authors’ response:** We are really grateful for your appreciation of our efforts. Besides, we have tried our best to address all your comments point by point as detailed below.

**Reviewer comment 1:** You need to put the Ethiopian guidelines regarding birth spacing in a wider context in the introduction. Most high-income countries have much shorter birth spacing. Why does Ethiopia (and a lot of other low and middle income countries) recommend a minimum of 3 years? It would benefit the reader a lot if you described the reasoning.

**A**uthors’ response: Definitely! Description of why does Ethiopia recommend a minimum of 3 years of birth spacing in a wider context in the introduction would benefit the reader a lot for easy understanding. Therefore, the following quoted text is added to the introduction section of the revised version manuscript as it can be seen by the yellow highlighted text on pages 3 and 4, under background section.

“Ethiopia had high population size as it was projected to reach more than 100 million and 4.0 total fertility rates in 2015. The country had also higher estimated pregnancy-related mortality ratio (PRM) of 412 deaths per 100,000 live births. Moreover, 1 in every 35 children dies within the first month; 1 in every 21 children dies before celebrating the first birthday; and 1 of every 15 children dies before reaching the fifth birthday (16). Therefore, the Ethiopian Federal Ministry of Health (FOMH) recommends spacing of childbirth at intervals of three to five years to reduce maternal, perinatal and infant mortality by optimizing the fertility rate in the country.

**Reviewer comment** 2) Please describe and justify the sampling frame in more detail. You should also provide the response rates for cases and controls. There should also be a figure 1 showing exactly how many cases and controls were recruited as opposed to the number included in the analysis.

**A**uthors’ response: **What a comment of relevance!** It would help increase understandability of the sampling procedure! Thus, the following detail is given.

“Multi stage sampling technique was employed to select the cases and controls. At first, 30% of the overall ‘*kebeles’* (three rural and five urban kebelles), were selected by simple random sampling technique. Then, for those rural *kebeles,* the authors first checked family folder from health extension workers. The family folder is an extension of the Ethiopian Community Health Information System (CHIS) at the most basic level of rural health system. Health extension workers (HEWs) make individualized household family member and assessment of each household’s health behavior and assign a set of health cards for individuals in each household. We reviewed the family folder of permanently residing women in each kebele that fulfilled the inclusion criteria (less than 3 years birth interval for cases and 3–5 years’ birth interval (including 3 and 5 years for controls) by registering the birth date of the last two successive children in a family with their corresponding household identification number. However, for urban ‘*kebeles’,* house to house visit (census) was conducted to identify permanently residing women that fulfilled the inclusion criteria (cases and controls) by registering the birth date of the last two successive children in a family with their corresponding household identification number. Using the respective household identification number, a sampling frame of the households containing cases and controls was prepared for each kebele. Then, proportional allocation of sample size was employed to determine the study participants from each kebele. Finally, cases and controls were selected by simple random sampling technique from the existing sampling frame. Whenever more than one eligible woman was found in same selected household, only one woman was chosen by lottery method. Thus, a sample of 678 women (226 cases and 452 controls) was recruited from the sampling frame for the study. But, from these recruited 678 women, 654 women (218 cases and 436 controls) agreed to be interviewed, thereby making a response rate of 96.5% (Figure 1)”.

Randomly selected

415 eligible women (Cases=134; Controls=281)

263 eligible women (Cases=92; Controls=171)

By referring to family folder

By census (house to house visit)

Eligible permanent dweller women (sampling frame)

Eligible permanent dweller women (Sampling frame)

Randomly selected

Five urban kebelles

Three rural *kebelles*

Overall kebelles in Dessie City Administration [**N**=18 urban and 8 rural kebeles]

Amendment is located on pages 5 and 6 , methods section, subsection of sample size determination and sampling procedure in the revised version manuscript as shown by the yellow highlighted text.

**Reviewer comment:**3) For your power calculations, you have not described how prevalent you estimated the relevant predictors of short inter-pregnancy interval to be? Some of the predictors you considered are very rare and you are not adequately powered to evaluate them. You should specify the minimum prevalence of the predictors you were powered to detect in relation the estimated minimum effect size.

**Authors’ response:** Yes indeed! For our power calculations, we have not described how prevalent we estimated the relevant predictors of short inter-pregnancy interval to be. Besides, as you said, some of the predictors we considered are rare and we are not adequately powered to evaluate them. Therefore, taking this comment and several exposure variables into account, we calculated the respective sample size just by considering the assumption of case to control ratio of 1: 2; CI: 95%; Power: 80%; minimum detectable AOR =2; design effect of 1.5 and 5% non-respondent rate. We selected the factor ‘contraceptive use’ because it yielded the maximum sample size as given in the following table (Table 1). Therefore, the sample size for this study was 678 (226 cases and 452 controls).

Table 1: Sample size determination using different factors in the literature and the respective assumptions using Open EPI INFO version 7 software.

| Factors | Assumption | Total sample size | References |
| --- | --- | --- | --- |
| Contraceptive user | P of exposure in controls =66.7% | 678 | (Hailu and Gulte, 2016) |
| Residence/urban | P of exposure in controls =52.1% | 540 | (Yohannes *et al.*, 2011) |
| Husbands’ occupation /Employee | P of exposure in controls =51.7% | 537 | (Yohannes *et al.*, 2011) |
| Mothers’ education /Has formal education | P of exposure in controls =48.3% | 524 | (Hailu and Gulte, 2016) |
| Parity />=5 children | P of exposure in controls = 49.2% | 524 | (Begna Z. *et al.*, 2013) |
| Sex of the index child /male | P of exposure in controls = 64.2% | 638 | (Begna Z. *et al.*, 2013) |
| Age of the mother/ 25-29 | P of exposure in controls = 24.9% | 576 | (Begna Z. *et al.*, 2013) |
| Status of index child /Alive | P of exposure in controls = 41.3% | 509 | (Tsegaye Dereje *et al.*, 2017) |
| Wealth index/ Richest | P of exposure in controls = 25.2% | 509 | (Hailu and Gulte, 2016) |

Amendment is located on pages 5 and 6 in the revised version manuscript as shown by the yellow highlighted text.

**Reviewer comment** 4) How did you decide what background factors to explore? What informed the questions that you initially decided to ask the study participants?

**A**uthors’ response: After reviewing different literature (Japheth Osotsi Awiti, 2013; Hailu and Gulte, 2016; Ayanaw A., 2008; Baschieri and Hinde, 2007, Begna Z. *et al.*, 2013; Central Statistical Agency (CSA) [Ethiopia] and ICF, 2016; Hailu and Gulte, 2016; Tsegaye Dereje *et al.*, 2017; Yohannes *et al.*, 2011) that addressed proximate, intermediate, socio- demographic and economic determinants of birth interval, we decided the background factors to be explored in this study.

**Reviewer comment** 5) Was there really no missing information for any of the covariates? If there was any missing information in any of the covariates, how was this dealt this? I can´t see that this is described in the methods. If you have any missing data, this should be dealt with using multiple imputations.

**Authors’ response**: There is no missing information for any of the covariates in this study. This was because incomplete questionnaires were returned to the data collectors for completion by referring to the respective household identification number on a daily basis of checking all the questionnaires.

Amendment is located on page 7, methods section, subsection of data quality control, in the revised version manuscript as shown by the yellow highlighted text.

**Reviewer comment** 6) Were any of the women included in the study related? I was wondering whether you have any dependencies in the data that should be dealt with in the regression analysis. For example by using robust standard errors.

**Authors’ response:** What a comment of paramount importance! Answering this comment helps assure data quality and management of bias. As mentioned in the sampling procedure, it was tried to minimize bias from intra-cluster correlation effect (dependencies) by selecting only one of the eligible women in a selected household. Besides, standard error was used during multivariate regressions and there was no any factor whose standard error greater than two indicating no dependency between mothers regarding the considered factors.

## Amendment is located on page 8, methods section, subsection of data processing and analysis, in the revised version manuscript as shown by the yellow highlighted text.

**Reviewer comment** 7) You should clearly show the p-values from the bivariate analyses in all tables (1-4). As far as I can tell, these bivariate analyses provided the basis for your decision for to carry some covariates forward to the regression analysis.

**A**uthors’ response: Undoubtedly! Considering your constructive comment, we have now clearly shown the p-values from the bivariate analyses in all tables (former tables 1-4, currently tables 2-5). As you said, these bivariate analyses provided the basis for our decision to carry some covariates forward to the regression analysis. The detailed response is given below and also shown by the yellow highlighted column of the P-value for tables 2-5.

**Table 2**: Socio-demographic characteristics on short birth interval among ever married mothers (case=218, control=436) in Dessie city administration, Dessie , Ethiopia 2019.

Amendment is located on page , line of the revised version manuscript as shown by the yellow highlighted text.

| Factors | Category | Case (%) | Control(%) | P value |
| --- | --- | --- | --- | --- |
| Rsidence | Urban | 130(59.7%) | 273(62.65) | 0.460 |
|  | Rural | 88(40.3%) | 163(37.4%) |  |
| Marital status | Married | 186(85.3%) | 364(83.5%) | 0.759 |
|  | Divorced | 21(9.6%) | 44(10.1%) |  |
|  | Widowed | 11(5.1%) | 28(6.4%) |  |
| Religion | Orthodox | 92(42.2%) | 173(39.7%) | 0.287 |
|  | Muslim | 124(56.9%) | 249(57.1%) |  |
|  | Protestant | 2(0.9%) | 14(3.2%) |  |
| Ethinicity | Amhara | 200(91.7%) | 399(91.5%) | 0.926 |
|  | Tgrai | 7(3.2%) | 11(2.5%) |  |
|  | Oromo | 6(2.7%) | 14(3.2%) |  |
|  | Others^1^ | 5(2.3%) | 12(2.8%) |  |
| Mother’s education | No formal education | 45(20.6%) | 70(16.1%) | 0.546 |
|  | read and write | 42(19.3%) | 86(19.7%) |  |
|  | Elementary | 34(15.6%) | 81(18.6%) |  |
|  | Secondary | 39(17.9%) | 89(20.4%) |  |
|  | Collage and above | 58(26.6%) | 110(25.2%) |  |
| Husband education | No formal education | 50(22.9%) | 69(15.8%) | 0.104 |
|  | read and write | 32(14.7%) | 69(15.8%) |  |
|  | Elementary | 13(5.9%) | 42(9.6%) |  |
|  | Secondary | 41(18.8%) | 72(16.5%) |  |
|  | College and above | 82(37.6%) | 184(42.2%) |  |
| Mothers’ occupation | employee(GO/NGO) | 43(19.7%) | 91(20.9%) | 0.730 |
|  | house wife | 125(57.3%) | 232(53.2%) |  |
|  | Merchant | 28(12.8%) | 53(12.2%) |  |
|  | Student | 9(4.1%) | 29(6.7%) |  |
|  | Farmer | 10(4.6%) | 19(4.4%) |  |
|  | daily workers | 3(1.4%) | 11(2.5%) |  |
|  | Others^2^ | 0(0%) | 1(0.2%) |  |
| Husband occupation | employee(GO/NGO) | 84(38.5%) | 164(37.6%) | 0.086 |
|  | Merchant | 66(30.3%) | 129(29.6%) |  |
|  | Student | 0(0%) | 2(0.5%) |  |
|  | Farmer | 63(28.9%) | 107(24.5%) |  |
|  | daily workers | 4(1.8%) | 23(5.3%) |  |
|  | Others^3^ | 1(0.5%) | 11(2.5%) |  |
| Number of wives  wealth index | One | 216(99.1%) | 434(99.5%) | 0.478 |
|  | More than one | 2(0.9%) | 2(0.5%) |  |
|  | Poorest | 57(26.1%) | 84(19.3%) | 0.096 |
|  | Second | 35(16.1%) | 80(18.3%) |  |
|  | Middle | 47(26.6%) | 83(19.0%) |  |
|  | Fourth | 42(19.3%) | 82(18.8%) |  |
|  | Richest | 37(17.0%) | 107(24.5%) |  |

^1^Afar, Gurage^2^ House servant,^3^Religious leader

**Table 3**: Knowledge and attitude of birth interval among ever married reproductive age mothers (case=218, control=436) in Dessie city administration, Dessie, Ethiopia 2019.

| Factors | Category | Case (%) | Control (%) | P value |
| --- | --- | --- | --- | --- |
|  |  |  |  |  |
| Heard about optimal birth interval | Yes | 165(75.7%) | 352(80.7%) | 0.336 |
|  | No | 53(24.3%) | 84(19.3%) |  |
| Optimum number of years between two successive births | Below three years | 19(11.5) | 46(13.1%) | 0.701 |
|  | Three to five years | 130(78.8%) | 280(79.5%) |  |
|  | Above five years | 13(7.8%) | 23(6.5%) |  |
|  | I am not sure | 3(1.8%) | 3(0.8%) | 0.562 |
| A minimum of 3 years of birth interval is essential between two successive births | Strongly agree | 81(37.2%) | 139(31.9%) |  |
|  | Agree | 134(61.5%) | 291(66.7%) |  |
|  | no idea | 2(0.9%) | 3(0.7%) |  |
|  | Disagree | 1(0.5%) | 3(0.7%) |  |
| Husband's perception regarding birth spacing | Disagree strongly | 28(12.8%) | 27(6.2%) | 0.001 |
|  | don't mind | 57(26.1%) | 152(34.9%) |  |
|  | Encouraging | 120(55.04%) | 246(56.4%) |  |
|  | Unknown | 13(5.96%) | 11(2.5%) |  |
| External influences to give birth in short interval | My family | 37(16.97%) | 61(13.99%) | 0.258 |
|  | Mother in law | 21(9.63%) | 60(13.76%) |  |
|  | Father in law | 7(3.2%) | 12(2.75%) |  |
|  | Societies norm | 9(4.1%) | 5(1.1%) |  |
|  | None | 144(66.1%) | 298(68.4%) |  |
| Perceived advantages of optimum birth spacing | Yes | 205(94.04%) | 406(93.1%) | 0.655 |
|  | No | 13(5.96%) | 30(6.9%) |  |
| Perceived disadvantages of short birth interval | Yes | 204(93.6%) | 404(92.7%) | 0.665 |
|  | No | 14(6.4%) | 32(7.3%) |  |

Table 4: Obstetrics related factors of short birth interval among ever married reproductive age mothers (case=218, control=436)inDessie city administration, Dessie, Ethiopia 2019.

| Factors | Category | Case (%) | Control (%) | P value |
| --- | --- | --- | --- | --- |
|  |  |  |  |  |
| Fetal outcome of first delivery | Live birth | 191(87.6%) | 382(87.62%) | 0.352 |
|  | still birth | 11(5.04%) | 13(2.98%) |  |
|  | Abortion | 3(1.4%) | 13(2.98%) |  |
|  | Neonatal mortality | 13(5.96%) | 28(6.42%) |  |
| Prior history of infertility | Yes | 4(1.83%) | 3(0.69%) | 0.279 |
|  | No | 214(98.17%) | 433(99.31%) |  |
| Ever given birth to any child who died | Yes | 31(14.2%) | 58(13.3%) | 0.723 |
|  | No | 187(85.8%) | 378(86.7%) |  |
| Male to female ratio of living children | More than one | 71(32.6%) | 160(36.7%) | 0.355 |
|  | One | 63(28.89%) | 135(30.96%) |  |
|  | Less than one | 49(22.48%) | 74(16.97%) |  |
|  | Males only | 15(6.9%) | 36(8.26%) |  |
|  | Females only | 20(9.17%) | 31(7.11%) |  |
| Previous to last pregnancy is planned | Yes | 180(82.6%) | 402(92.2%) | 0.001 |
|  | No | 38(17.4%) | 34(7.8%) |  |
| Practice postpartum abstinence before the last child | Yes | 161(73.85%) | 359(82.3%) | 0.011 |
|  | No | 57(26.15%) | 77(17.7%) |  |
| Mode of delivery of previous to last birth | Vaginal delivery | 197(90.4%) | 392(89.9%) | 0.981 |
|  | Cesarean section | 14(6.4%) | 29(6.7%) |  |
|  | Instrumental delivery | 7(3.2%) | 15(3.4%) |  |
| ANC follow up in preceding pregnancy | Yes | 172(78.9%) | 387(88.8%) | 0.009 |
|  | No | 46(21.1%) | 49(11.2%) |  |
| Place of delivery of previous to last birth | Home | 25(11.5%) | 39(8.9%) | 0.308 |
|  | Health institution | 193(88.5%) | 397(91.1%) |  |
| Pattern of menstruation in previous to last deliveries | Regular | 185(84.9%) | 362(83.02%) | 0.550 |
|  | Irregular | 33(15.1%) | 74(16.97%) |  |
| Ever had chronic diseases (HTN,DM ,others) before the last child | Yes | 16(7.3%) | 44(10.1%) | 0.255 |
|  | No | 202(92.7%) | 392(89.9%) |  |
| Ever had history of postpartum complications in previous to last deliveries | Yes | 26(11.9%) | 61(13.99%) | 0.464 |
|  | No | 192(88.1%) | 375(86.01%) |  |
| Last child sex | Male | 121(55.5%) | 238(54.6%) | 0.824 |
|  | Female | 97(44.5%) | 198(45.4%) |  |
| Is last child alive | Yes | 217(99.5%) | 434(99.5%) | 0.741 |
|  | No | 1(0.5%) | 2(0.5%) |  |
| previous to last child sex | Male | 72(33%) | 235(53.9%) | 0.001 |
|  | Female | 116(53.2%) | 201(46.1%) |  |
| Is previous to last child alive | Yes | 215(98.6%) | 434(99.5%) | 0.254 |
|  | No | 3(1.4%) | 2(0.5%) |  |
| Parity | <5 | 180 (82.5%) | 370(84.8%) | 0.450 |
|  | >=5 | 38(17.5%) | 66(15.2%) |  |

Table 5: Breast feeding duration and contraceptive use among ever married reproductive age mothers in Dessie city administration, Dessie, Ethiopia 2019

| Factors | **Category** | **Case (%)** | **Control(%)** | | **P value** |
| --- | --- | --- | --- | --- | --- |
| Did you breast feed previous to last child | Yes | 152(69.7%) | 400(91.7%) | | 0.001 |
|  | No | 66(30.3%) | 36(8.3%) | |  |
| Did you exclusively breastfeed previous to last child | Yes | 80(52.6%) | 295(73.8%) | | 0.001 |
|  | No | 72(47.4%) | 105(26.2) | |  |
| Breast feeding duration | 0-11 | 134(61.5%) | 61(13.99%) | | 0.001 |
|  | 12-23 | 64(29.4%) | 197(45.18%) | |  |
|  | >=24 | 20(9.2%) | 178(40.83%) | |  |
| Using any of the modern methods before the conception of your last child | Yes | 98(44.95%) | 411(94.3%) | | 0.001 |
|  | No | 120(55.05%) | 25(5.7%) | |  |
| Decision maker about Family planning | Self | 104(47.7%) | 190(43.58) | |  |
|  | Both husband and wife | 97(44.5%) | 227(52.06%) | | 0.261 |
|  | Husband only | 3(1.4%) | 13(2.98%) | |  |
|  | No one | 14(6.4%) | 6(1.38%) | |  |
| Perception of family planning method | Agree | 183(83.9%) | 428(98.2%) | | 0.001 |
|  | Disagree | 34(15.6%) | 4(0.9%) | |  |
|  | Neutral | 1(0.5%) | 4(0.9%) | |  |
| Distance from health institution | Less than 30 minutes | 93(42.7%) | 197(45.2%) | 0.799 | |
|  | 30-1hrs | 123(56.4%) | 236(54.1%) |  | |
|  | Greater than 1 hr | 2(0.9%) | 3(0.7%) |  | |

**Reviewer comment** 8) You used a backwards approach to your variable selection, if I am interpreting the methods correctly. Were the results similar if you used a forward or a stepwise variable selection procedure?

**A**uthors’ response: We used backward stepwise LR to identify variables which had the largest contribution to the model. The result in forward or a stepwise variable selection method was similar on significance of the variables, but little change in adjusted odds ratio, p value and confidence interval were observed.

Amendment is located on page 14, results section, subsection of determinants of short birth interval as shown by the yellow highlighted text in the revised version manuscript.

**Reviewer comment** 9) I would recommend that you have a native English speaker help you look through the manuscript.

**A**uthors’ response: what a similar comment with reviewer two. Therefore, similar response is given as mentioned below in the quoted text.

“From repeated proof-reading of the manuscript, we found several grammatical errors, interlinings, police titles, punctuation errors, wordings and spelling errors. Therefore, finding our colleague who has Master of Arts in English, we have tried our best to thoroughly copyedit the manuscript for English language usage. These changes are found throughout the revised version manuscript.”

**Dear reviewer #2**

*After going through the entire manuscript, you forwarded your constructive comments which we missed to touch. Therefore, we are glad enough to express our sincerest thanks for your in-depth review and comments that could help improve the tone of our paper.*

**Reviewer suggestion**: This was an interesting manuscript and I enjoyed reading your manuscript. However, the authors should consider addressing the following

**A**uthors’ response: We are really grateful for your appreciation of our efforts. Besides, we have tried our best to address all your comments point by point as detailed below.

**Reviewer comment** 1: The entire manuscript needs to be revised for grammatical errors and punctuation issues. For example, on page 2, the conclusion section of the abstract, the authors started a sentence with a lower case alphabet. Also, on page 3 (introduction) the first sentences need revisiting as we express maternal mortality per 100,000 live births and not "live birth". Similarly, on page 3, the last sentence in paragraph 1 needs revising particularly the sentence ...."the problem is still major public concern."

**A**uthors’ response: Sure! From repeated proof-reading of the manuscript, we found several grammatical errors, interlinings, police titles, punctuation errors, wordings and spelling errors. Therefore, finding our colleague who has Master of Arts in English, we have tried our best to thoroughly copyedit the manuscript for English language usage. These changes are found throughout the revised version manuscript. Moreover, the aforementioned reviewer’s specific concerns are addressed as listed below.

**Conclusion:** Contraceptive use, breast feeding duration, age at first birth, preceding child sex and knowing the duration of birth interval correctly were independent determinants of short birthinterval.

Inter birth interval refers to the time interval from one child’s birth date until the next child’s birth date between two consecutive live births.

Amendment is located on pages 2 and 3 of the revised version manuscript as shown by the yellow highlighted text.

**Reviewer comment** 2. The authors stated on page 3 that ...."national guideline for family planning services according to the Ethiopian FMOH’s recommendation" I will recommend that the authors should provide a sentence or two from this guidelines that are applicable/relevant to their study.

**A**uthors’ response: Considering the given comment, the following sentence was taken from the Ethiopian national guideline of family planning services.

“The Ethiopian Federal Ministry of Health (FOMH) recommends spacing of childbirth at intervals of three to five years to reduce maternal, perinatal and infant mortality by optimizing the fertility rate in the country.”

Amendment is located on page 3, paragraph 3 of the revised version manuscript as shown by the yellow highlighted text.

**Reviewer comment** 3. The authors need to state the aim in the last paragraph of their introduction. At present, this is not really clear.

**A**uthors’ response: Absolutely! At present, the aim of this study is not really clear. Therefore, it is now stated in the last paragraph of the introduction concisely as shown by the yellow highlighted text on page 4 of the revised version manuscript. The aim is also given below.

“Therefore, this study was aimed at identifying factors that have significant odds of association with short inter-birth interval among a community-based sample of Ethiopian women in Dessie city administration, 2019.”

**Reviewer comment** 4. On page 8, Table 1, the authors need to correct the word 'college' which is wrongly written as 'collage'. Additionally the word 'widowed' is also wrongly written as 'windowed'

**A**uthors’ response: Certainly! The misspelt words are corrected accordingly as shown by the yellow highlighted text in table 2 of the revised version manuscript which is also given below.

**Table 2**: Socio-demographic characteristics on short birth interval among ever married mothers (case=218, control=436) in Dessie city administration, Dessie , Ethiopia 2019

| Factors | Category | Case (%) | Control(%) |
| --- | --- | --- | --- |
| Rsidence | Urban | 130(59.7%) | 273(62.65) |
|  | Rural | 88(40.3%) | 163(37.4%) |
| Marital status | Married | 186(85.3%) | 364(83.5%) |
|  | Divorced | 21(9.6%) | 44(10.1%) |
|  | Widowed | 11(5.1%) | 28(6.4%) |
| Religion | Orthodox | 92(42.2%) | 173(39.7%) |
|  | Muslim | 124(56.9%) | 249(57.1%) |
|  | Protestant | 2(0.9%) | 14(3.2%) |
| Ethinicity | Amhara | 200(91.7%) | 399(91.5%) |
|  | Tgrai | 7(3.2%) | 11(2.5%) |
|  | Oromo | 6(2.7%) | 14(3.2%) |
|  | Others^1^ | 5(2.3%) | 12(2.8%) |
| Mother’s education | No formal education | 45(20.6%) | 70(16.1%) |
|  | read and write | 42(19.3%) | 86(19.7%) |
|  | Elementary | 34(15.6%) | 81(18.6%) |
|  | Secondary | 39(17.9%) | 89(20.4%) |
|  | Collage and above | 58(26.6%) | 110(25.2%) |
| Husband education | No formal education | 50(22.9%) | 69(15.8%) |
|  | read and write | 32(14.7%) | 69(15.8%) |
|  | Elementary | 13(5.9%) | 42(9.6%) |
|  | Secondary | 41(18.8%) | 72(16.5%) |
|  | College and above | 82(37.6%) | 184(42.2%) |
| Mothers’ occupation | employee(GO/NGO) | 43(19.7%) | 91(20.9%) |
|  | house wife | 125(57.3%) | 232(53.2%) |
|  | Merchant | 28(12.8%) | 53(12.2%) |
|  | Student | 9(4.1%) | 29(6.7%) |
|  | Farmer | 10(4.6%) | 19(4.4%) |
|  | daily workers | 3(1.4%) | 11(2.5%) |
|  | Others^2^ | 0(0%) | 1(0.2%) |
| Husband occupation | employee(GO/NGO) | 84(38.5%) | 164(37.6%) |
|  | Merchant | 66(30.3%) | 129(29.6%) |
|  | Student | 0(0%) | 2(0.5%) |
|  | Farmer | 63(28.9%) | 107(24.5%) |
|  | daily workers | 4(1.8%) | 23(5.3%) |
|  | Others^3^ | 1(0.5%) | 11(2.5%) |
| Number of wives  wealth index | One | 216(99.1%) | 434(99.5%) |
|  | More than one | 2(0.9%) | 2(0.5%) |
|  | Poorest | 57(26.1%) | 84(19.3%) |
|  | Second | 35(16.1%) | 80(18.3%) |
|  | Middle | 47(26.6%) | 83(19.0%) |
|  | Fourth | 42(19.3%) | 82(18.8%) |
|  | Richest | 37(17.0%) | 107(24.5%) |

^1^Afar, Gurage^2^ House servant,^3^Religious leader

Amendment is located on page 9, in table 2 , results section, sociodemographic characteristics subsection of the revised version manuscript as shown by the yellow highlighted text.

**Reviewer comment** 5. On page 14, Table 5, the authors need to double-check the p-values as there as selected p-values <0.05 that were not highlighted.

**A**uthors’ response: Based on the given comment, Table 5 (currently, table 6) has been double-checked if there is any P-value <0.05 that were not highlighted. Thus, the double-checked table is given below with yellow highlight of P-values that were not highlighted.

**Table 6:** Multivariable analysis on the determinants of short birth interval among ever married reproductive age mothers in Dessie city administration, Dessie, Ethiopia 2019.

| Factors | | | Case | | Controls | | Crude OR(95% CI) | | p-value | | AOR(95%CI) | p-value |
| --- | --- | --- | --- | --- | --- | --- | --- | --- | --- | --- | --- | --- |
| Preceding pregnancy was planned  yes  no | | | 180  38 | | 402  34 | | 0.401(0.244-0.657)  1 | | .001 | | 0.800 (.348-1.839)  1 | .599 |
| practice of postpartum abstinence in preceding child  yes  no | | | 161  57 | | 359  77 | | 0.606(0.410-0.8941)  1 | | .012 | | 0.875(0.482-1.587)  1 | .659 |
| ANC follow up in preceding pregnancy  Yes  No | | | 177  41 | | 387  49 | | 0.547(0.348-0.858)  1 | | .009 | | 0.895(0.400-2.003)  1 | 0.787 |
| breast fed duration from previous to last child  0-11  12-23  >=24 | | | 134  64  20 | | 61  197  178 | | 1  0.148(0.098-0.224)  0.051(0.029-0.089) | | .001  .001 | | **1**  **0.291(0.154-0.550)**  **0.098(0.047-0.208)** | **.001***  **.001*** |
| previous to Last child sex  male  female | | | 72  146 | | 235  201 | | 0.422(0.300-0.592)  1 | | 0.01 | | **0.463(0.282-0.761**)  1 | **.002*** |
| using any of the modern methods before the conception of your last child  yes  no | | | 98  120 | | 411  25 | | 1  20.1(12.407-32.662) | | .001 | | 1  **11.221(5.953-21.151)** | .**001*** |
| knowledge to appropriate duration of birth interval  correctly know  not correctly know | | | 130  88 | | 280  156 | | 0.823(.589-1.149)  1 | | 0.253 | | **0.446(0.245-0.811)**  **1** | **.008*** |
| Husband education  No formal education  Had formal education | | | 82  136 | | 138  298 | | 1.302(0.926-1.830)  1 | | 0.129 | | 1.236 (0.633-2.416)  1 | .535 |
| age at first marriage  less than 18  18-25  Greater than 25 | | | 24  154  40 | | 82  328  26 | | 1  1.604(0.979-2.628)  5.256(2.68-10.286) | | 0.061  .001 | | 1  1.148(0.550-2.398)  0.478(0.113-2.024) | .713  .316 |
| age at first birth (years)  less than 28  >=28 | | | 160  58 | | 413  23 | | 0.154(0.092-0.257)  1 | | 0.001 | | **0.363(0.166-0.793)**  **1** | **0.011*** |
| no of living children  0-2  3-4  >=5 | | | 55  125  38 | | 90  280  66 | | 1  0.731(0.492-1.086)  0.942(0.559-1.587) | | 0.120  0.823 | | 1  .617(0.338-1.124)  1.109(0.489-2.514) | .115  .696 |
| Husband perception to birth spacing  Disagree strongly  Dont mind  Encouraging  Unknown | | | 28  57  120  13 | | 27  152  246  11 | | 1  0.362(0.196-0.666)  0.470(0.266-0.833)  1.140(0.436-2.980) | | 0.001  0.010  0.790 | | 1  0.376(0.136-1.036)  0.557(0.221-1.401)  0.873(0.195-3.908) | .059  .214  .859 |
| Wealth index  Poorest  Second  Middle  Fourth | | | 57  35  47  42 | | 84  80  83  82 | | 1.962(1.187-3.245)  1.265(0.733-2.183)  1.638(0.976-2.747)  1.481(0.874-2.510) | | .009.  0.398  .062  .144 | | 2.012(0.872-4.645)  1.486(0.606-3.647)  2.378(1.086-5.210  1.823(0.780-4.262) | .101  .387  .030  0.166 |
| Richest | | | 37 | | 107 | | 1 | |  | |  |  |
|  |  |  | |  | |  | |  | |  |  |  |

***for Significant association at p<0.05)**

Amendment is located in table 6, on pages 15 and 16 of the revised version manuscript as shown by the yellow highlighted text.
**Reviewer comment** 6: On page 15, paragraph 2, the authors should correct the word 'consistency' which was misspelt as 'consistence'.

**A**uthors’ response: Yes indeed! On page 15, paragraph 2 of the original document, the word 'consistency' was misspelt as 'consistence'. Thus, it has been corrected as listed below.

“The consistency could be due to the fact that contraceptive use contributes to birth spacing thereby reducing the total fertility rate by different mechanisms on normal reproductive process [25].”

Amendment is located on page 17, first paragraph of the revised version manuscript as shown by the yellow highlighted text.

**Reviewer comment** 7. The limitations of the study (page 17) needs revision and should be reported before the conclusion. Specifically, there was no mention of how the recall and social desirability bias reported in the manuscript were dealt with.

**A**uthors’ response: Quite sure! The limitations of the study (page 17 of the original document), is currently revised and reported before the conclusion. Specifically, a mention of how the recall and social desirability bias reported in the manuscript were dealt with is stated clearly as given below.

“The recall bias was dealt with enabling mothers attach their children’s birth dates to unforgettable Ethiopian holidays and calendar days. Besides, it was tried to minimize social desirability bias by conducting probed maternal interviews of the events (factors) by the trained data collectors.”

Amendment is located on 18, strength and limitation section of the revised version manuscript as shown by the yellow highlighted text.

**Reviewer comment 8.** The authors also need to provide a few sentences on the key strength of their study.

**A**uthors’ response: Certainly! The key strength of our study was “Using community based unmatched case control study design, high response rate and inclusion of both urban and rural communities.”

Amendment is located on page 18, strength and limitation section of the revised version manuscript as shown by the yellow highlighted text.

***We look forward to receiving your immediate and kind response!***

***Habtamu Shimelis Hailemeskel (On behalf of all authors)***
