# Supplementary material for: Determinants of short birth interval among ever married reproductive age women: A community based unmatched case control study at Dessie city administration, Northern Ethiopia
Source: PLoS One. 2020 Dec 4;15(12):e0243046. doi: 10.1371/journal.pone.0243046 (PMC7717527; doi:10.1371/journal.pone.0243046)
Supplement: Attachment — Submitted filename: Response letter (Revised 2 version).docx [file pone.0243046.s003.docx]

**Dear academic Editor (Sharon Mary Brownie)**

*After going through the entire revised version manuscript, you forwarded your constructive editorial comment. Therefore, we are glad enough to express our sincerest thanks for your helpful comment that could help improve the tone and readability of our paper.*

**Editor comment:** Thank you for submitting your manuscript to PLOS ONE. After careful consideration, we feel that it has merit but does not fully meet PLOS ONE’s publication criteria as it currently stands. Therefore, we invite you to submit a revised version of the manuscript that addresses the points raised during the review process.

**Authors’ response:** We are really delighted with your constructive editorial comments. Hence, we have addressed the points raised during the review process and the corrections are incorporated within the second round revised version manuscript. All the improved changes are shown by tracked insertions and deletions.

**Dear reviewer 1**

*After going through the entire manuscript, you forwarded your constructive comments which we missed to touch. Therefore, we are glad enough to express our sincerest thanks for your in-depth review and comments that could help improve the tone of our paper.*

Reviewer comment: The authors have addressed most of my comments. I only have a few minor points.

**Authors’ response:** We are most grateful for your acknowledgment of our efforts in addressing most of your comments. Besides, we have tried to address your current concerns point by point as detailed below.

Reviewer comment: 1) Delete this text from the conclusion:

"We want to emphasize that our study involved neither experimental nor observational design and hence our recommendations are not based on causal mechanisms. Our recommendations are
rather based on the assumption that short birth interval is a potentially modifiable risk factor of
adverse pregnancy outcomes. Hence, intervening on its identified independent predictors
(significant factors) helps optimize inter-birth interval."

Your study is observational and this suggested deleted text does not add any useful information.
**Authors’ response:** We strongly agree with the reviewer’s comment and hence the aforementioned text has been deleted from the conclusion section as it can be appreciated from the tracked deletion, paragraph 1 of the conclusion, on page 19 of the revised version manuscript.

Reviewer comment: 2) Move the last paragraph away from the conclusion section to the discussion:

"Based on our findings, local health care providers (physicians, midwives, nurses and health
extension workers), the city health department and policy makers should focus on different
strategies for creating parental awareness about the importance of modern contraceptive use,
being primiparous before 28 years old and maternal knowledge of birth spacing. Moreover, we
strongly recommend that mothers should prolong their breastfeeding practice for at least two
years because its effect for optimizing birth interval has been witnessed by many other studies,
WHO and UNICEF. However, encouraging breast feeding up to two years may not warrant a
reduction of birth interval because increasing breast feeding duration merely does not increase
period of amenorrhea. This could in turn be due to differences among maternal breastfeeding
practices, maternal age and parity. Women who are partially breast-feeding are at higher risk of
conceiving than women who are fully breast-feeding. The period of lactational amenorrhoea
tends to be longer for older and multiparous than for younger and primiparous women. Besides,
regardless of their breastfeeding practices, the other possible independent factor that may affect
lactational infertility is maternal nutritional status. Therefore, despite the aforementioned
confounders, maternal practice of optimal breastfeeding helps them optimize not only their
health but also feto-neonatal and childhood survival."

**Authors’ response:** Well! We are grateful for your comment of importance. Thus, the last paragraph has been moved away from the conclusion section to the discussion as shown by the tracked insertions and deletions in the discussion and conclusion sections of the revised version manuscript on pages 18 and 19.

Reviewer comment: 3) You can consider shortening the section on the sample size determination and selection procedure, and the section on data quality control.

**Authors’ response:** Great thanks! Based on the given comment, the section on the sample size determination and selection procedure, and the section on data quality control have been shortened to a reasonable extent. The amendment can be appreciated from the tracked deletion on pages 5, 6 and 7, in the methods section, subsection of sample size determination and selection procedure and data quality control.
